# A rice calcium-dependent protein kinase OsCPK9 positively regulates drought stress tolerance and spikelet fertility

**DOI:** 10.1186/1471-2229-14-133

**Published:** 2014-05-17

**Authors:** Shuya Wei, Wei Hu, Xiaomin Deng, Yingying Zhang, Xiaodong Liu, Xudong Zhao, Qingchen Luo, Zhengyi Jin, Yin Li, Shiyi Zhou, Tao Sun, Lianzhe Wang, Guangxiao Yang, Guangyuan He

**Affiliations:** 1 The Genetic Engineering International Cooperation Base of Chinese Ministry of Science and Technology, Key Laboratory of Molecular Biophysics of Chinese Ministry of Education, College of Life Science and Technology, Huazhong University of Science & Technology, Wuhan 430074, China; 2 Present address: Institute of Tropical Bioscience and Biotechnology, Chinese Academy of Tropical Agricultural Sciences, Haikou 571101, China

**Keywords:** Abscisic acid (ABA) signaling, Abiotic stresses, Calcium-dependent protein kinase (CDPK), Drought stress tolerance, Rice, Spikelet fertility

## Abstract

**Background:**

In plants, calcium-dependent protein kinases (CDPKs) are involved in tolerance to abiotic stresses and in plant seed development. However, the functions of only a few rice CDPKs have been clarified. At present, it is unclear whether CDPKs also play a role in regulating spikelet fertility.

**Results:**

We cloned and characterized the rice *CDPK* gene, *OsCPK9. OsCPK9* transcription was induced by abscisic acid (ABA), PEG6000, and NaCl treatments. The results of *OsCPK9* overexpression (*OsCPK9-*OX) and *OsCPK9* RNA interference (*OsCPK9*-RNAi) analyses revealed that *OsCPK9* plays a positive role in drought stress tolerance and spikelet fertility. Physiological analyses revealed that *OsCPK9* improves drought stress tolerance by enhancing stomatal closure and by improving the osmotic adjustment ability of the plant. It also improves pollen viability, thereby increasing spikelet fertility. In *OsCPK9*-OX plants, shoot and root elongation showed enhanced sensitivity to ABA, compared with that of wild-type. Overexpression and RNA interference of *OsCPK9* affected the transcript levels of ABA- and stress-responsive genes.

**Conclusions:**

Our results demonstrated that *OsCPK9* is a positive regulator of abiotic stress tolerance, spikelet fertility, and ABA sensitivity.

## Background

Calcium, as a second messenger, plays important roles in a variety of signal transduction pathways. Several classes of calcium-sensing proteins, including calcium-dependent protein kinases (CDPKs), calcineurin B-like (CBL) proteins, and calmodulin (CaM), have been characterized in plants [1]. CDPKs activated by Ca^2+^ and modulate downstream targets of calcium signaling in plants [2-4]. CDPKs participate in stress signaling transduction pathways through either stimulus-dependent activation or directed functional target protein phosphorylation [2,3,5-7].

Genome-wide analyses have identified 34 *CDPK* genes in Arabidopsis [8,9]. Some Arabidopsis *CDPKs* have been reported to be involved in abiotic stress responses and abscisic acid (ABA) signaling. Loss-of-function mutants of *CPK4* and *CPK11* showed decreased tolerance to salt and drought stresses, and ABA-insensitive phenotypes for seed germination, seedling growth, and stomatal movement. CPK4 and CPK11 phosphorylate two ABA-responsive transcription factors, ABF1 and ABF4 to mediate the ABA signaling pathway [10]. *CPK6-*overexpressing plants showed enhanced tolerance to salt and drought stresses and *cpk3* mutants exhibited a salt-sensitive phenotype [11,12]. CPK3 and CPK6 also function in controlling of ABA-regulated stomatal signaling and guard cell ion channels. ABA-induced stomatal closure was partially impaired in a *cpk3/cpk6* mutant [13]. CPK6 activates the slow anion channel (SLAC1) and CPK3 activates SLAC1 as well as its guard cell homolog SLAH3. These activations are calcium-dependent and are controlled by the ABA signaling component phosphatase ABI1 [14,15]. CPK32 phosphorylates the ABA-responsive transcription factor ABF4 *in vitro*, and *CPK32*-overexpressing plants displayed increased sensitivity to ABA during seeds germination as a result of up-regulated expressions of genes controlled by ABF4 [16]. *CPK10*-overexpression and T-DNA insertion mutant analyses have shown that *CPK10* is involved in drought stress tolerance. Moreover, CPK10, through its interaction with heat shock protein 1 (HSP1), plays a role in ABA- and Ca^2+^-mediated regulation of stomatal movement [17]. Together, these studies have shown that Arabidopsis CPK family members can positively regulate abiotic stress tolerance and ABA signaling.

However, Arabidopsis *CPK23-*overexpressing lines showed a drought- and salt-sensitive phenotype and increased stomatal aperture. Accordingly, *cpk23* mutants showed improved tolerance to drought and salt stresses and reduced stomatal aperture [18]. Arabidopsis seedlings with a loss-of-function of *CPK21* also showed increased tolerance to hyperosmotic stress [19]. CPK21 and CPK23 were shown to control the activation state of SLAC1 in Ca^2+^-independent manner [20]. Arabidopsis *CPK12-*RNAi lines were hypersensitive to ABA during seed germination and root elongation [21]. The results of these studies suggested that some Arabidopsis CPKs function as negative regulators of abiotic stress tolerance and ABA signaling. Therefore, the experimental evidences indicate that CDPK-mediated abiotic stress and ABA responses are complex in Arabidopsis.

Although 31 *CDPK* genes have been identified in the rice genome [22,23], the functions of only a few have been explored so far. For example, *OsCDPK7-*overexpressing plants exhibited increased resistance to cold, drought, and salinity stresses [24]. *OsCPK21* was shown to be involved in increasing ABA sensitivity and conferring salt stress tolerance. Compared with wild-type, *OsCPK21*-overexpressing plants showed a higher survival rate under salt stress and a stronger inhibition of seedling growth by ABA [25]. *OsCPK12* overexpression and *OsCPK12* RNA interference analyses revealed that OsCPK12 positively regulates rice tolerance to salt stress by controlling the expression of *OsAPx2*, *OsAPx8* and *OsrbohI*. Moreover, *OsCPK12*-overexpressing lines showed increased sensitivity to ABA and enhanced susceptibility to blast fungus, probably because of decreased production of reactive oxygen species and/or the involvement of OsCPK12 in the ABA signaling pathway [26].

The calcium-dependent seed-specific protein kinase (SPK) is a key regulator of seed development. SPK is involved in regulating the metabolic pathway responsible for the conversion of sucrose into storage starch in immature seeds [27]. *OsCDPK1* negatively regulates the expressions of enzymes required for GA biosynthesis and seed size, but positively regulates drought stress tolerance through the14-3-3 protein [28]. However, it is unclear whether CDPKs play a role in regulating spikelet fertility. Spikelet fertility that is affected by anther dehiscence, pollen production and the number of germinating pollen grains on the stigma is an important component of yield [29-31]. In the present research, *OsCPK9* overexpression (*OsCPK9-*OX) and interference (*OsCPK9-*RNAi) analyses indicate that *OsCPK9* positively regulates abiotic stress tolerance, spikelet fertility, and ABA sensitivity. These findings contribute to our understanding of CDPK-mediated abiotic stress responses and ABA signaling, and will be useful for improving the stress tolerance and quality of rice.

## Results

### Expression patterns of *OsCPK9* in rice

**Figure 1:**
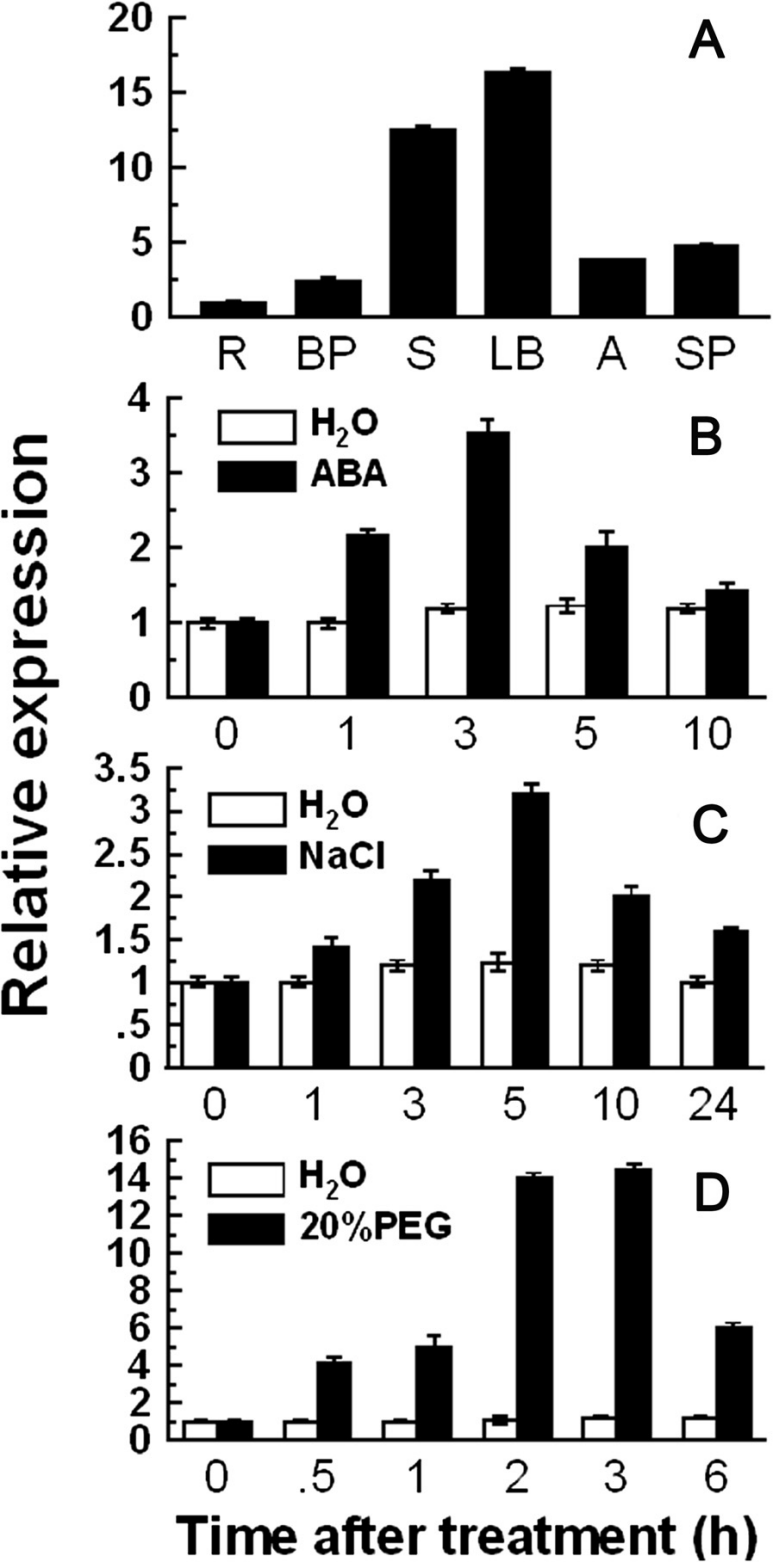
**qRT-PCR analysis of *OsCPK9 *expression in different organs (A) and in rice leaves after 100 μM ABA (B), 200 mM NaCl (C), or 20% PEG6000 (D) treatments.** R: root; BP: basal part; S: stem; LB: leaf blade; A: anther; SP: spikelet. The mRNA fold difference is relative to that of root samples for **(A)** or distilled water-treated samples at 0 h for **(B, C and D)**. Data are means ± SE of three independent experiments.

To investigate the *OsCPK9* expression patterns in different rice organs, we conducted quantitative reverse transcription-polymerase chain reaction (qRT-PCR) analyses using mRNA isolated from various organs as the templates. *OsCPK9* transcripts present in all organs tested including the root, basal part, stem, leaf blade, anther, and spikelet, with higher transcript levels in the leaf blade and stem than in other organs (Figure 1A). To detect the transcriptional response of *OsCPK9* to abiotic stresses and ABA, various treatments were applied to rice plants. After ABA treatment, the expression of *OsCPK9* increased at 1 h and reached the highest level at 3 h followed by a decrease (Figure 1B). *OsCPK9* transcription was also induced to the highest level at 5 h and 2 h after NaCl and PEG6000 treatments respectively (Figure 1C; 1D). Therefore, *OsCPK9* transcription was up-regulated by ABA, NaCl, and PEG6000 treatments in comparison to control, implying its function in the responses to abiotic stresses and ABA.

### Generation of *OsCPK9* transgenic rice lines

To further study the function of *OsCPK9 in planta*, we generated *OsCPK9*-OX (OE) and *OsCPK9*-RNAi (Ri) transgenic lines. The RT-PCR results showed that the transcript levels of *OsCPK9* were markedly higher in *OsCPK9*-OX lines than in wild type (WT) with the highest transcriptional levels of *OsCPK9* in OE28 (Additional file 1: Figure S1). In contrast, the transcript levels of *OsCPK9* were reduced in *OsCPK9*-RNAi lines, with the lowest transcript levels of *OsCPK9* in Ri2 (Additional file 1: Figure S1). We detected the intron sequence introduced into the construct, confirming the presence of the construct in *OsCPK9*-RNAi lines (Additional file 1: Figure S1). These results confirmed that *OsCPK9*-OX and *OsCPK9*-RNAi transgenic lines were successfully produced.

### OsCPK9 increases plants’ tolerance to drought, osmotic, and dehydration stresses

**Figure 2:**
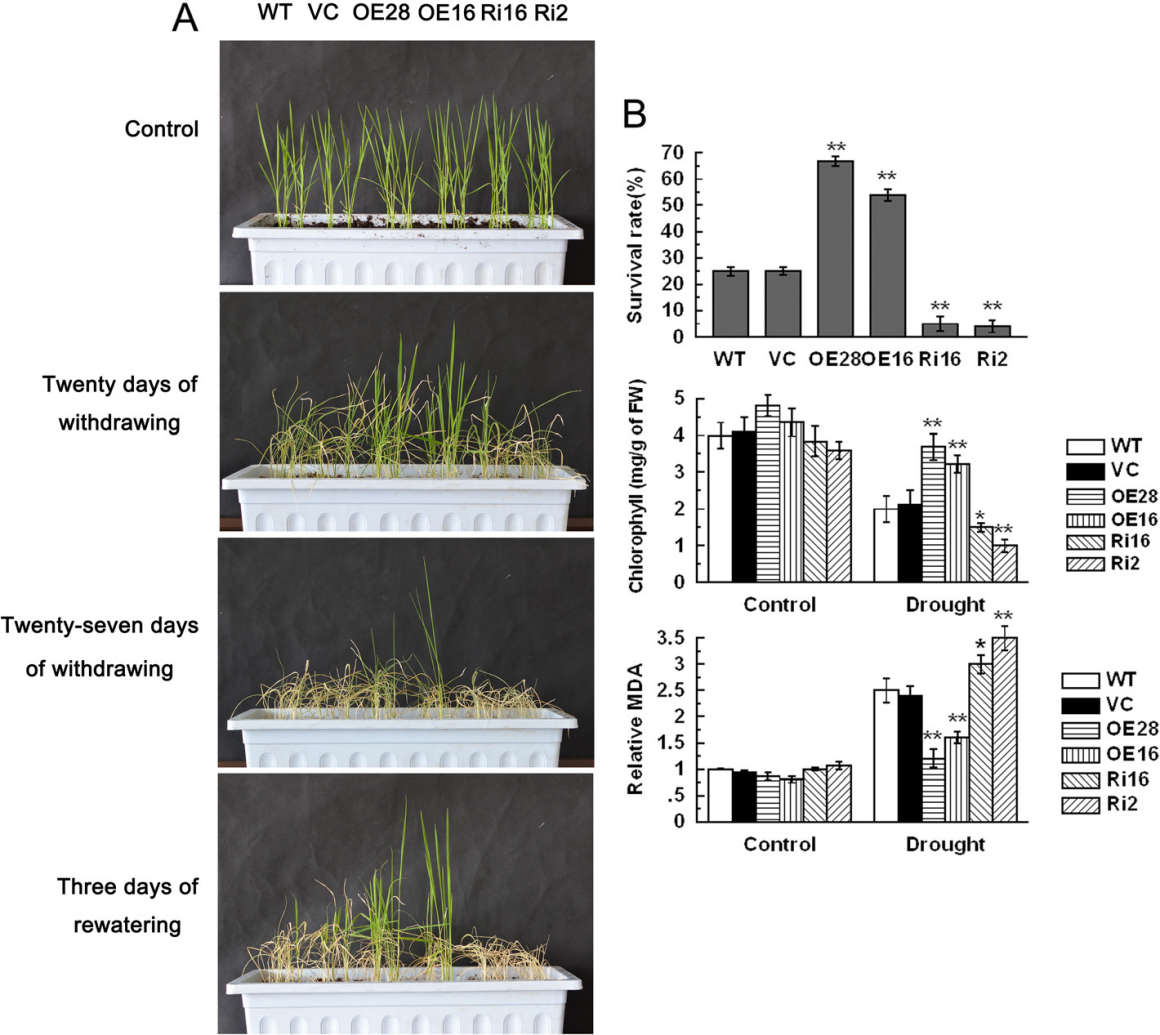
**Drought stress tolerance of *OsCPK9*-OX and *OsCPK9*-RNAi transgenic lines. (A)** Photographs of transgenic lines and controls after drought treatment. Three-week-old rice seedlings were deprived of water for 20 or 27 days, followed by 3 days of recovery. Photos of transgenic lines and controls were taken at these time points. **(B)** Survival rates, chlorophyll, and MDA content of transgenic lines and controls with or without drought treatment. Three-week-old rice seedlings were deprived of water for 27 days, followed by 3 days recovery, then survival rates were calculated. Three-week-old rice seedlings were deprived of water for 15 days and then chlorophyll, and MDA content were measured in leaf samples. Data are means ± SE of four independent experiments. Asterisks indicate significant difference between WT and transgenic lines (*p <0.05; **p <0.01).

To investigate the drought stress tolerance of *OsCPK9*-OX and *OsCPK9*-RNAi lines, 3-week-old rice seedlings were subjected to a drought treatment. After 20 or 27 days of drought, *OsCPK9*-OX lines grew well. In contrast, the growth of the *OsCPK9*-RNAi lines was inhibited compared with that of control (Figure 2A). After 27 days of drought and 3 days of recovery, the survival rates of *OsCPK9*-OX lines OE28 and OE16 (67% and 54% respectively) were higher than that of WT (25%), while *OsCPK9*-RNAi lines Ri16 and Ri2 showed very low survival rates (5% and 4% respectively) (Figure 2A; 2B). Although there were no significant differences in chlorophyll and malondialdehyde (MDA) contents between controls and transgenic lines under normal growth conditions, clear differences were observed between control and transgenic lines after the drought treatment. The chlorophyll content was higher in *OsCPK9*-OX lines, but lower in *OsCPK9*-RNAi lines compared with that in the control after drought treatment (Figure 2B). The MDA content was lower in *OsCPK9*-OX lines, but higher in *OsCPK9*-RNAi lines, compared with that in the control after drought treatment (Figure 2B). These results indicated that *OsCPK9* plays a positive role in drought stress tolerance.

To determine the osmotic stress tolerance of *OsCPK9*-OX and *OsCPK9*-RNAi lines, 2-week-old rice seedlings were treated with 20% PEG6000 for 8 h and followed with 1, 2, or 7 days of recovery. At different treatment stages, the *OsCPK9*-OX lines showed better growth than that of controls, and the *OsCPK9*-RNAi lines showed worse growth (Additional file 1: Figure S2A). After the 8 h osmotic treatment, *OsCPK9*-OX plants showed a lower MDA content and higher soluble sugars and proline contents, while *OsCPK9*-RNAi plants showed a higher MDA content and lower soluble sugars and proline contents, compared with those of wild type (WT) and the vector control (VC) (Additional file 1: Figure S2B). After 7 days of recovery, compared with controls, *OsCPK9*-OX plants had higher biomass, reflected by longer roots and shoots, greater fresh weight, less wilted leaves, and more green leaves. In contrast, the biomass of *OsCPK9*-RNAi plants was lower than that of control plants (Additional file 1: Table S3). These analyses of physiological indices confirmed that osmotic stress tolerance is increased in *OsCPK9*-OX lines and decreased in *OsCPK9*-RNAi lines.

To analyze the dehydration stress tolerance of *OsCPK9*-OX and *OsCPK9*-RNAi lines, 2-week-old rice seedlings were exposed to air. *OsCPK9*-OX lines tolerated a 5 h dehydration treatment (Additional file 1: Figure S3). After a 10 days recovery, *OsCPK9*-OX lines grew more robustly than did WT and VC, as reflected by their longer roots and shoots and greater fresh weight (Additional file 1: Figure S3; Additional file 1: Table S4). These results indicated that *OsCPK9*-OX plants have increased tolerance to dehydration stress.

### OsCPK9 functions in water retention by increasing proline and soluble sugars contents and improving stomatal closure under drought stress

**Figure 3:**
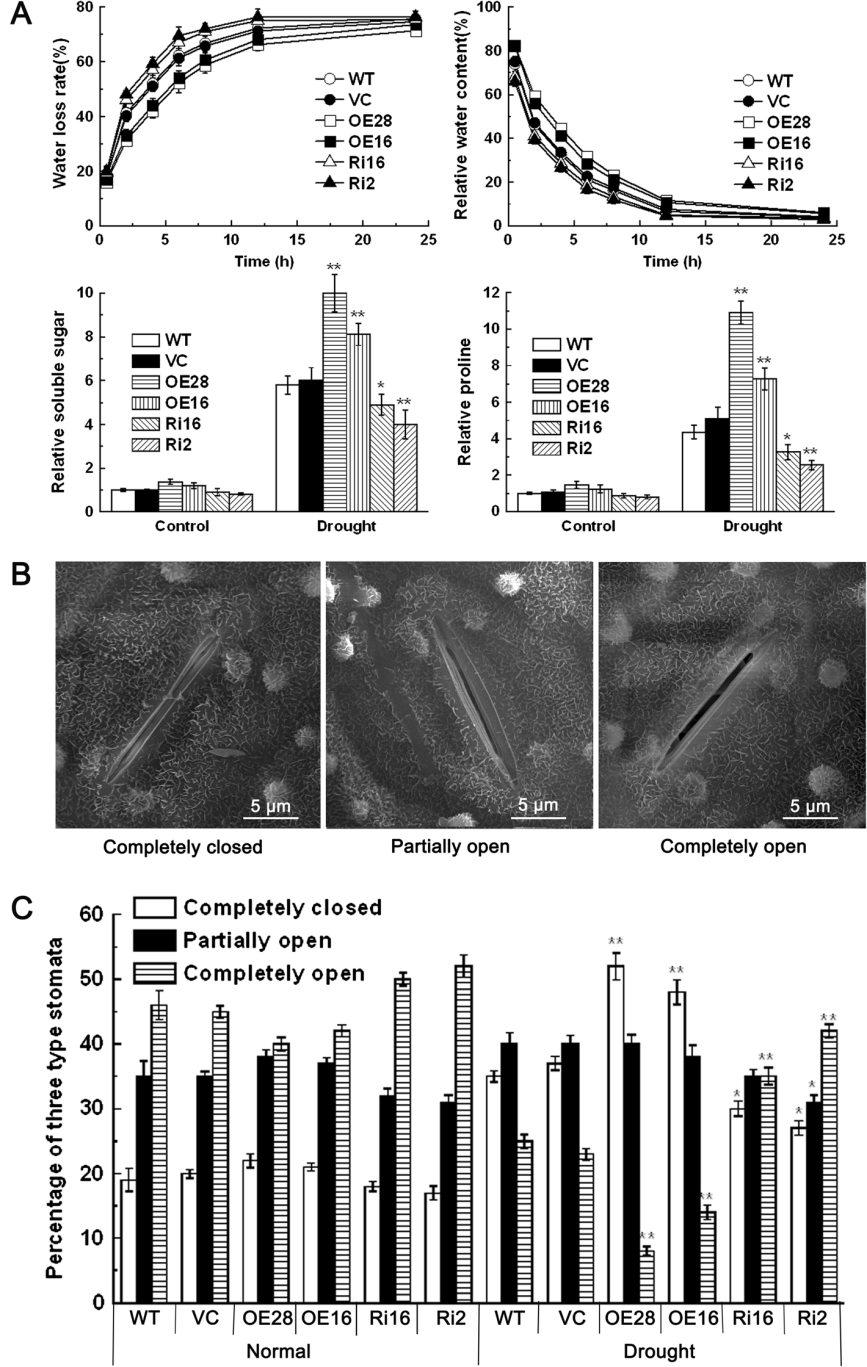
**WLR, RWC, soluble sugars, proline, and stomatal status of *OsCPK9*-OX and *OsCPK9*-RNAi transgenic lines. (A)** WLR, RWC, soluble sugars, and proline contents of *OsCPK9*-OX and *OsCPK9*-RNAi transgenic lines. **(B)** Scanning electron microscope images of stomatal status; open, closed, partially open. **(C)** Proportions of open, closed, and partially open stomata. Leaves of 3-week-old rice seedlings were collected to determine the WLR and RWC of control plants and transgenic lines. Three-week-old rice seedlings were deprived of water for 15 days and then soluble sugars, proline and stomatal status were examined with leaf samples. Data are means ± SE of four independent experiments for (A) and three independent experiments for (C). Asterisks indicate significant difference between the WT and transgenic lines (*p <0.05; **p <0.01).

Plants with high capacity for water retention can better survive drought or dehydration stress. During 0 to 25 hours of a dehydration treatment, *OsCPK9*-OX lines retained a high relative water content (RWC) and showed a low water loss rate (WLR), while *OsCPK9*-RNAi lines had lower RWC and higher WLR compared with those of WT and VC (Figure 3A). These results indicated that *OsCPK9* plays a positive role in improving the ability of the plant to retain water under dehydration conditions.

Osmotic adjustment and stomatal closure are the main physiological mechanisms to reduce water loss under dehydration or drought conditions in plants. To elucidate the physiological mechanism by which *OsCPK9* confers tolerance to drought and dehydration stresses and improves the ability of plant to retain water, we quantified osmolytes (proline and soluble sugars) in *OsCPK9*-OX and *OsCPK9*-RNAi lines. Under normal growth conditions, there were no significant differences between controls and transgenic lines in terms of their proline and soluble sugars contents (Figure 3A). Under drought conditions, *OsCPK9*-OX lines accumulated larger amounts of proline and soluble sugars, but *OsCPK9*-RNAi lines accumulated smaller amounts of proline and soluble sugars, compared with those in controls (Figure 3A). Additionally, the status of stomata was observed and counted in controls and transgenic lines. Under normal growth conditions, there were no significant differences in stomatal status between controls and transgenic lines. After the drought treatment, 35% and 37% of stomata were completely closed in WT and VC plants, respectively, while greater proportions of stomata were closed in *OsCPK9*-OX lines (52% in OE28 and 48% in OE16). Accordingly, there were smaller proportions of completely opened stomata in *OsCPK9*-OX lines, but larger proportions in *OsCPK9*-RNAi lines (Figure 3B; 3C; Additional file 1: Table S5). There was a slightly lower proportion of partially opened stomata in *OsCPK9*-RNAi lines than in controls. These results indicated that *OsCPK9* affects osmotic balance and stomatal movement under drought conditions.

### OsCPK9 improves pollen maturation and spikelet fertility under normal conditions

**Figure 4:**
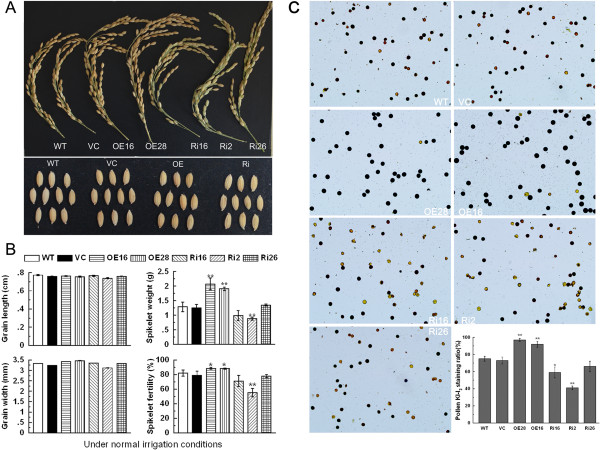
**Spikelet fertility and mature pollen viability of transgenic lines and WT under normal conditions.** Photographs of mature spikelets harvested from control plants and transgenic lines were taken **(A)**. Grain length, grain width, spikelet weight, and spikelet fertility of control plants and transgenic lines **(B)**. Mature pollen grains from control plants and transgenic lines were stained by I_2_-KI **(C)**. Data are means ± SE calculated from four independent experiments. Asterisks indicate significant difference between WT and transgenic lines (*p <0.05; **p <0.01).

We harvested and analyzed spikelets to evaluate the grain development in the transgenic lines under normal conditions. Spikelet weight is 1.29 g and spikelet fertility is 81.88% in WT rice plants. *OsCPK9*-OX lines had greater spikelet weight (OE16 2.07 g; OE28 1.90 g) and spikelet fertility (OE16 88.45%; OE28 88.19%), compared with those of controls. In contrast, the spikelets of *OsCPK9*-RNAi lines were less fertile (Ri16 71.24%; Ri2 55.36%) and had a smaller spikelet weight (Ri16 0.98 g; Ri2 0.87 g) than those of WT and VC lines. There was no obvious difference in grain length and width between WT and transgenic lines (Figure 4A; Figure 4B). Therefore, spikelet weight and spikelet fertility of rice were correlated with the expression of *OsCPK9*. Because the number of mature pollen is an important impact factor of spikelet fertility, we further investigate pollen status of control plants and transgenic lines using I_2_-KI staining. The results indicated that *OsCPK9*-OX lines had a higher mature pollen staining ratio, while *OsCPK9*-RNAi lines had a lower ratio than those of WT and VC (Figure 4C). Mature pollen staining ratio reflects pollen viability. The mature pollen staining ratio correlated with the expression of *OsCPK9* suggested that *OsCPK9* functions in increasing pollen viability. Collectively, these results indicated that *OsCPK9* enhances spikelet fertility by regulating pollen maturation.

### Responses of *OsCPK9*-OX and *OsCPK9*-RNAi lines to ABA

**Figure 5:**
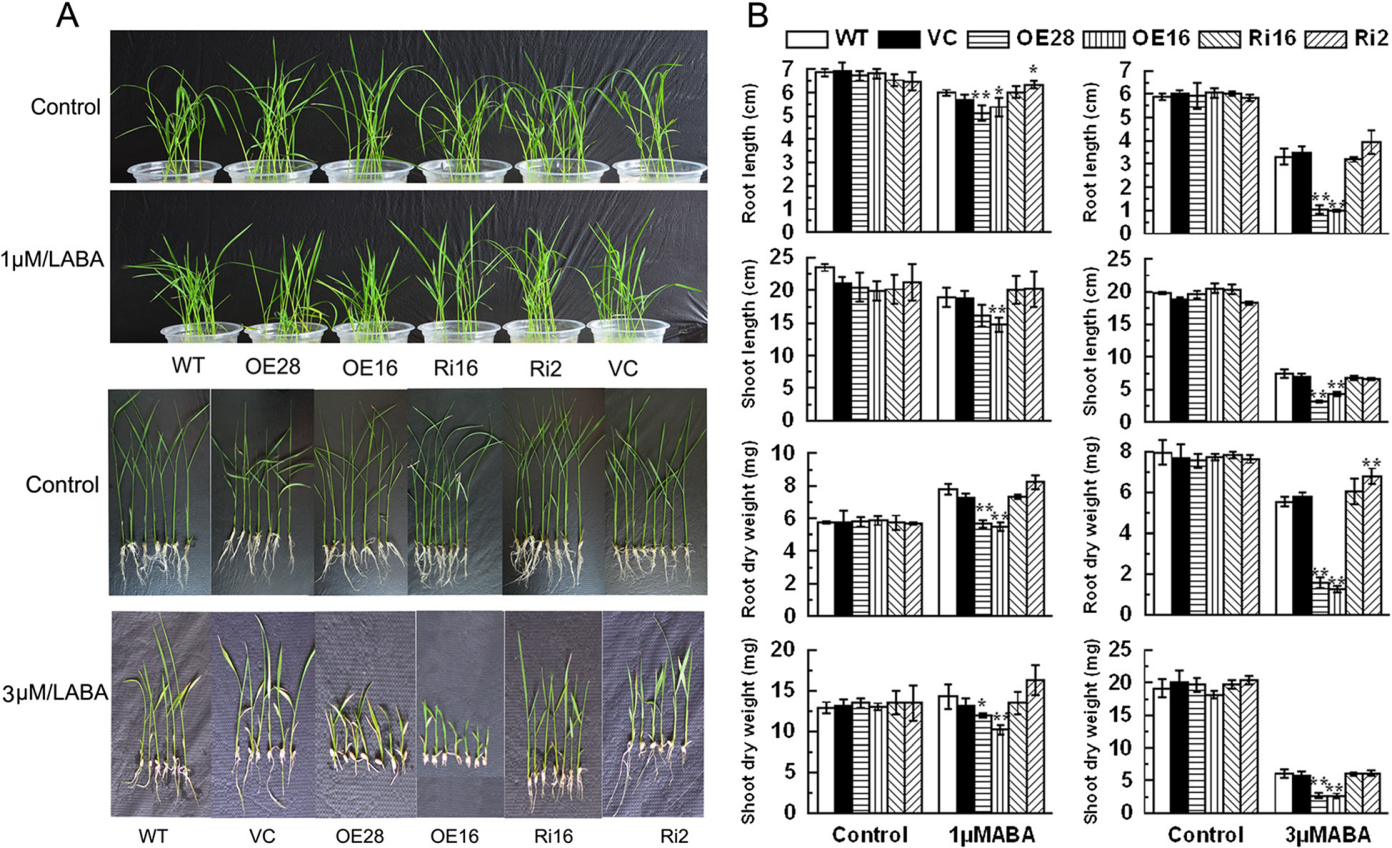
**ABA sensitivity of *OsCPK9*-OX and *OsCPK9*-RNAi rice lines.** Three-day-old rice seedlings were treated with 1 μM or 3 μM ABA for 14 days and then photographed **(A)**. Length and dry weight of roots and shoots of rice seedlings harvested after the 14-day ABA treatment **(B)**. Data are means ± SE calculated from four independent experiments. Asterisks indicate significant difference between the WT and transgenic lines (*p <0.05; **p <0.01).

To explore whether *OsCPK9* is involved in the ABA signaling response, *OsCPK9*-OX and *OsCPK9*-RNAi lines were treated with exogenous ABA. Under 1 μM ABA treatment, *OsCPK9*-OX lines showed shorter roots and shoots and lower root and shoot dry weights than those of WT and VC (Figure 5; Additional file 1: Table S6). Although seedlings growth of control and transgenic plants was inhibited by a 3 μM ABA treatment, it was more strongly inhibited in *OsCPK9*-OX plants than in WT and VC plants (Figure 5A). The 3 μM ABA treatment had a stronger negative effect on root length, shoot length, and root and shoot dry weights of *OsCPK9*-OX plants than on those parameters in WT and VC plants (Figure 5B; Additional file 1: Table S7). Conversely, ABA did not significantly affect seedling growth and root elongation of *OsCPK9*-RNAi lines, compared with that of control plants after ABA treatment. These results confirmed that *OsCPK9*-OX lines are more sensitive to ABA than WT and VC.

### OsCPK9 regulates ABA- and stress-responsive genes under osmotic stress and ABA treatment

**Figure 6:**
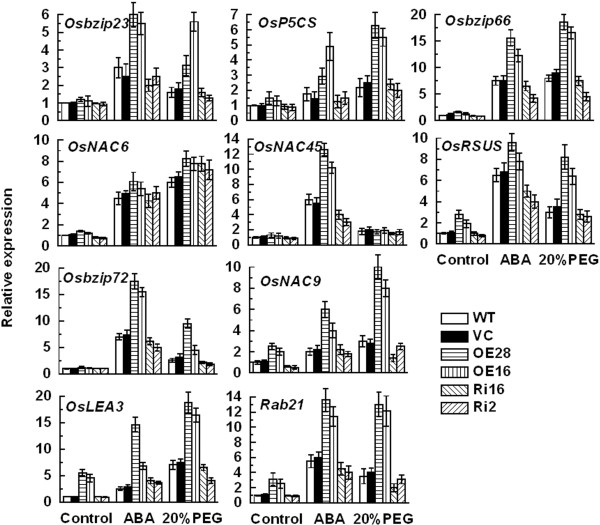
**Expression analysis of selected ABA- and stress-responsive genes in *OsCPK9*-OX, *OsCPK9-*RNAi, and control lines under no stress, ABA, or PEG6000 treatments.** Three-day-old rice seedlings were treated with 1 μM ABA for 14 days. Two-week-old rice seedlings were treated without (normal conditions) or with 20% PEG6000 for 8 h. Leaves were collected to detect transcript levels of those ABA- and stress-responsive genes. The mRNA fold difference is relative to that of WT samples under normal conditions. Data are means ± SE of three independent experiments.

To gain a deeper understanding of *OsCPK9* function in osmotic stress tolerance and the ABA response, we analyzed the transcript levels of some selected ABA- and stress-responsive genes by qRT-PCR analysis in control and transgenic lines under normal conditions, osmotic stress, and ABA treatment (Figure 6). The following genes were selected for analysis: *Rab21*, which encodes a basic glycine-rich protein [32]; *OsLEA3*, encoding a late embryogenesis abundant protein [33]; *OsP5CS*, encoding Δ^1^-pyrroline-5-carboxylate synthetase, which is involved in proline biosynthesis [34]; *OsNAC6*, *OsNAC9* and *OsNAC45*, which encode NAC-type transcription factors [35-38]; *OsRSUS*, encoding sucrose synthase [27] and *Osbzip23*, *Osbzip66*, and *Osbzip72*, which encode ABF-type transcription factors [39-41]. Under normal conditions, the transcript levels of *OsNAC9* were higher in *OsCPK9*-OX lines and lower in *OsCPK9*-RNAi lines, compared with that in WT. The transcript levels of *OsLEA3*, *Rab21*, *OsRSUS,* and *OsP5CS* were higher in *OsCPK9*-OX lines than in WT and VC. After ABA treatment, the transcript levels of *Rab21*, *Osbzip66*, *OsNAC45*, and *OsRSUS* were higher in *OsCPK9*-OX but lower in *OsCPK9*-RNAi lines, compared with their respective levels in WT and VC. The transcript levels of *Osbzip23*, *OsLEA3*, *OsP5CS*, *OsNAC9* and *Osbzip72* were higher in *OsCPK9*-OX than in WT and VC. Under PEG6000 treatment, the transcript levels of all of the selected genes except for *OsNAC6* and *OsNAC45* were higher in *OsCPK9*-OX plants than in the control. The transcript levels of the tested genes were confirmed by RT-PCR, and the results were generally consistent with those detected by qRT-PCR analysis (Additional file 1: Figure S4). These results suggested that *OsCPK9* expression affects the transcription of ABA- and stress-associated genes.

## Discussion

### OsCPK9 plays a positive role in drought, osmotic, and dehydration stress responses

*OsCPK9* belongs to the group III-b *CDPK* family [22]. The *OsCPK9* gene contains five exons and four introns. The OsCPK9 protein is composed of 574 amino acid residues with a predicted relative molecular mass of 63.9 kDa. It has a protein kinase domain, a calmodulin-like domain with four conserved EF-hand motifs, an autoinhibitory junction domain, and an N-terminal variable region [22]. It also has potential N-terminal myristoylation and palmitoylation sites [22]. Previously, *OsCPK9* expression in response to abiotic stresses was examined using a cDNA microarray. The results showed that *OsCPK9* was induced by salt and desiccation treatments [23]. In this study, *OsCPK9* transcription was induced by a PEG6000 treatment, implying that OsCPK9 also functions in the osmotic stress response (Figure 1). To assess the role of *OsCPK9* under drought conditions, we engineered rice lines in which *OsCPK9* was overexpressed or knocked down. Our results suggested that OsCPK9 is a positive regulator of the responses to drought, osmotic, and dehydration stresses (Figure 2; Additional file 1: Figure S2 and S3). These results are consistent with those of previous studies on some other *CDPK* genes that positively regulate drought stress tolerance [18,24,28].

### OsCPK9 confers tolerance to drought stress by improving osmotic adjustment and stomatal movement

The ability to retain water is crucial for plants to combat drought. Our results show that OsCPK9 is involved in maintaining the ability of plants to retain water, and hence, it confers drought stress tolerance (Figure 3A). We further explored the physiological mechanism by which OsCPK9 enables the plant to retain water. When water is limiting, plants accumulate compatible osmolytes such as soluble sugars and proline to decrease the cellular osmotic potential [42]. Our results showed that there were increased contents of both soluble sugars and proline in *OsCPK9*-OX lines, but decreased contents of these substances in *OsCPK9*-RNAi lines (Figure 3A). Thus, OsCPK9 functions in osmotic adjustment, improving the ability of the plant to retain water during drought. Also, stomatal movement controls not only CO_2_ uptake but also water loss to the atmosphere, thereby playing important roles in drought tolerance of crops [43]. Some CDPKs play vital roles in regulating stomatal movement. For example, overexpression of *ZmCPK4* resulted in increased ABA-mediated stomatal closure [44]. ABA- and Ca^2+^-induced stomatal closure were partially impaired in a *cpk3cpk6* mutant [13]. The Arabidopsis *CPK4* and *CPK11* genes were shown to be involved in ABA-regulated stomatal closure [10]. In the present study, *OsCPK9*-OX lines showed a significantly higher proportion of completely closed stomata under drought treatment, which may contribute to reduced water loss (Figure 3B and 3C). These results provided physiological evidence that OsCPK9 confers drought stress tolerance by enhancing the osmotic adjustment ability of the plant and by promoting stomatal closure, thereby reducing water loss.

### OsCPK9 regulates expression of stress-associated genes in response to drought

To gain a deeper understanding of the function of OsCPK9 under abiotic stresses, we analyzed the transcript levels of some stress-inducible genes. Under osmotic stress, the transcript levels of *Rab21*, *OsP5CS*, *OsLEA3*, *OsNAC9*, Osbzip23, *Osbzip66*, and *Osbzip72* were higher in *OsCPK9*-OX lines than in WT and VC (Figure 6). In previous studies, *Rab21* was shown to be induced by water stress, and overexpression of *OsP5CS*, *OsLEA3*, *OsNAC9*, *Osbzip23*, and *Osbzip72* enhanced tolerance to abiotic stresses [32,38,40,41,45,46]. It was also reported that transcript levels of some stress-responsive genes were higher in other *OsCPK*-overexpressing rice lines than in controls under abiotic stresses. The transcript levels of *OsLEA3*, *OsP5CS*, *Osbzip23*, and *OsNAC6* were higher in *OsCPK21*-FOX and *OsCPK13*-FOX plants than in WT plants under salt stress [25]. Similarly, *OsCDPK7*-overexpressing plants showed increased transcription of *OsLEA3* in roots after a salt treatment [24]. These results demonstrated that OsCPK9 is involved in increasing transcription of stress-associated genes, thereby improving tolerance to drought stress.

### OsCPK9 is involved in spikelet fertility

In a previous study, an analysis of *CDPK* gene family members revealed that transcripts for 23 genes deferentially accumulated during reproductive developmental stages [23]. In maize, a pollen-specific *CDPK* was only transcribed at the late stages of pollen development [47]. In petunia, *PiCDPK1* and *PiCDPK2* were involved in divergently regulating pollen tube growth. *PiCDPK1* played an important role in growth polarity, whereas *PiCDPK2* functioned in pollen tube extension [48]. These studies demonstrated that CDPKs function as important calcium sensors in pollen tube growth and seed development. However, it remained unknown whether CDPKs play a role in spikelet fertility. We detected *OsCPK9* transcript not only in vegetative organs, but also in two reproductive organs, anther and spikelet (Figure 1A). Further investigations suggested that OsCPK9 plays a role in increasing spikelet fertility (Figure 4A; 4B). Pollen viability reflected by mature pollen staining ratio plays an important role in spikelet fertility [49]. The mature pollen staining ratio determined by I_2_-KI staining was correlated with the expression of *OsCPK9*, indicating that *OsCPK9* positively regulates starch accumulation, pollen viability, and hence increases spikelet fertility (Figure 4C). The formation of mature and fertile pollen grains, taking place inside the anther, depends on supply of assimilates, in the form of sucrose, provided mainly by the leaves [50]. Starch biosynthesis during the final phases of pollen maturation is critical not only because starch provides a source of energy for pollen germination, but also because it is a checkpoint of pollen maturity [51]. The absence of starch deposition is a remarkable phenotype in male-sterile pollen [52]. Upregulation of *OsRSUS* in leaves of *OsCPK9*-overexpressing rice plants may increase sucrose supply to pollen for starch accumulation, therefore contributes to improved pollen viability and spikelet fertility (Figure 6). Whether OsCPK9 could directly influence starch accumulation in pollen needs further investigation.

### OsCPK9 possibly acts in an ABA-dependent manner

It is well established that the phytohormone ABA maintains seed dormancy and inhibits seed germination and seedling growth [53]. Drought induces ABA biosynthesis and triggers ABA-dependent signaling pathways [54]. Thus, we investigated the *OsCPK9* response to ABA. The *OsCPK9*-overexpressing lines were more sensitive to ABA than WT and VC (Figure 5). Arabidopsis CDPKs are involved in ABA signaling by phosphorylating basic leucine zipper class transcription factor proteins (bZIP). Arabidopsis CPK4 and CPK11 phosphorylate two bZIP factors, ABF1 and ABF4 [10]. Consistently, Arabidopsis CPK32 interacts with ABF4 and phosphorylates it *in vitro*[16]. Moreover, CPK4, CPK11, and CPK32 are involved in ABA-regulated physiological processes and abiotic stress tolerance [10,16]. Additionally, Osbzip66, Osbzip72, and Osbzip23 function in ABA signaling and/or abiotic stress tolerance [39-41,55,56]. The transcript levels of *Osbzip66*, *Osbzip72,* and *Osbzip23* increased in *OsCPK9*-OX lines under osmotic and ABA treatments (Figure 6). OsCPK9 may function with Osbzip66, Osbzip72, and Osbzip23 to mediate ABA signaling and abiotic stress responses. Furthermore, our results showed that OsCPK9 plays a positive role in regulating *Rab21*, *OsNAC9*, *OsLEA3*, and *OsP5CS* transcription under osmotic stress and ABA treatment (Figure 6). These genes are responsive to abiotic stresses and ABA signaling [57-60]. Therefore, the increased ABA sensitivity and higher transcript levels of ABA- and stress-responsive genes in *OsCPK9*-OX rice lines indicate that *OsCPK9* positively regulates abiotic stress tolerance in an ABA-dependent manner.

## Conclusions

We characterized the function of *OsCPK9*, a rice *CDPK* gene. *OsCPK9* overexpression and interference analyses revealed that *OsCPK9* positively regulates drought stress tolerance by enhancing stomatal closure and the osmotic adjustment ability of the plant. *OsCPK9* also improves pollen viability, thereby increasing spikelet fertility. The *OsCPK9*-OX rice lines exhibited increased sensitivity to ABA. These findings help to clarify details of the CDPK-mediated abiotic stress responses and the role of ABA signaling in improving stress tolerance and rice quality. In the future, identifying the direct targets of OsCPK9 would be useful to determine the molecular mechanism of CDPKs.

## Methods

### Plant materials and treatments

Rice (*Oryza sativa* L. cv. Nipponbare) seeds were germinated on MS agar medium and grown on hydroponic culture in a growth chamber (70% humidity, 14 h light/10 h dark cycle, 26°C) [61]. For *OsCPK9* expression assays under PEG6000, NaCl, and ABA treatments, rice seeds were germinated and grown for two weeks. Rice seedlings were then transferred into plastic boxes containing either 20% PEG6000, 200 mM NaCl, or 100 μM ABA for up to 24 h. A no treatment control was always included. Transcript levels of *OsCPK9* were detected in rice seedling leaves. To assess *OsCPK9* expression in different organs, root, basal part (30 mm) of seedling, stem, leaf blade, anther, and spikelet were collected from the rice plants.

### qRT-PCR analysis

qRT-PCR was employed to examine *OsCPK9* expression in different organs, in response to PEG6000, NaCl and ABA treatments, and for the expression of ABA- and stress-responsive genes. Primers (Additional file 1: Table S1) used in qRT-PCR showed high specificity, as determined by agarose gel electrophoresis and sequencing. In all experiments, appropriate negative controls without template were included to detect primer dimers and/or contamination. Prior to experiments, qRT-PCR was optimized through a series of template and primer dilutions. Amplification efficiencies for the internal control and target genes were between 0.92 and 1.14. Samples were run in triplicates and analyzed using the Opticon Monitor 2 qRT-PCR software. Expression levels of target genes were normalized to *OsActin* expression. Relative expression level of genes was calculated using the 2^–ΔΔCt^ formula [62].

### Plant transformation and transgenic plant generation

To construct the *OsCPK9*-OX vector, the coding sequence of *OsCPK9* was introduced into pCAMBIA1301 under CaMV 35S promoter control using primers P1 and P2 (Additional file 1: Table S2). To construct the *OsCPK9* RNAi vector, a 280 bp cDNA fragment encoding partial *OsCPK9* was included downstream of the CaMV 35S promoter in both sense and antisense orientations spaced by a 548 bp intron of wheat *TAK14* (Accession: AF325198) (Additional file 1: Table S2, P3-P8). These recombinant plasmids and vacant pCAMBIA1301 vector were introduced into *Agrobacterium tumefaciens* strain EHA105 to transform rice plants. Transgenic rice plants were generated using an Agrobacterium-mediated transformation method [63]. Seeds obtained from transgenic and vacant vector lines were selected on MS medium with 50 mg/L hygromycin. The hygromycin-resistant T_1_ seedlings were further examined by PCR analysis using primers to amplify *HYG* (Additional file 1: Table S2, P9 and P10). The homozygous T_2_*OsCPK9*-OX lines OE28 and OE16, *OsCPK9*-RNAi lines Ri16, Ri2 and Ri26, and VC line were used in further studies. *OsCPK9* expression in these T_2_ lines was detected by RT-PCR analysis using an *OsActin* control.

### Stress tolerance and ABA response analysis of WT and transgenic lines

For drought stress tolerance analysis, rice seeds were germinated on MS agar medium for 5 days and then grown in soil for 16 days in a growth chamber. Three-week-old rice seedlings were deprived of water for 27 days. This mimicked drought period was followed by a 3 days recovery. Survival rates were calculated (each sample contains 30 seedlings). Three-week-old rice seedlings were deprived of water for 15 days and then the chlorophyll, MDA, proline, soluble sugars, and status of stomata were examined by leaf samplings. Each sample represented four replicates (each replicate had 4-6 seedlings). For the osmotic stress tolerance assay, rice seeds germinated on MS agar medium for 5 days and then grown on hydroponic culture for 9 days in a growth chamber. Two-week-old rice seedlings with similar growth state were treated with 20% PEG6000 for 8 h. Seedlings were then allowed to recover for 7 days. After treatment with PEG6000 for 8 h, rice seedling leaves were sampled to detect soluble sugars, proline, MDA levels. Each sample represented four replicates (each replicate contained three leaves from the same position of independent plants). After recovery, root and shoot length, fresh weight, wilted and green leaves of rice seedlings were examined. Each sample represented four replicates (each replicate had 4-6 seedlings). For the dehydration stress tolerance assay, rice seeds were germinated on MS agar medium for 5 days and then grown on hydroponic culture for 9 days in a growth chamber. Two-week-old rice seedlings were placed on a bench in the growth chamber for 5 h, followed by recovery for 10 days. Rice seedlings were collected to examine root and shoot length and fresh weight. Each sample represented four replicates (each replicate had 4-6 seedlings). For the ABA sensitivity assay, rice seeds germinated on MS agar medium for 3 days, and then grown on hydroponic culture containing either 1 μM or 3 μM ABA for 14 days. After a 14 days treatment, rice seedlings were collected to examine the length and dry weight of root and shoot. Each sample again represented four replicates (each replicate had 6-7 seedlings).

### Physiological indices measurement

WLR was measured according to methods described by Zhang *et al*. [42]. Briefly, rice leaves were detached from seedlings and fresh weight (FW) was assessed. Detached leaves were placed on a bench at room temperature to induce dehydration and weighed at designated time intervals (desiccated weight). WLR (%) = (FW – desiccated weight)/FW × 100. RWC was measured according to Hu *et al*. [64]. The dehydrated leaves were soaked in distilled water for 4 h and turgid weight (TW) was recorded. Leaves were finally dried for 48 h at 80°C to obtain total dry weight (DW). RWC was calculated as follows: RWC (%) = [(desiccated weight – DW)/(TW– DW)] × 100. Proline was detected as described by Troll & Lindsley [65]. Soluble sugars was measured *via* a phenol–sulphuric acid method [66]. Chlorophyll content was determined by UV spectrophotometry [67], and MDA content measured *via* a method described by Heath & Packer [68]. Rice stomatal status was determined through scanning electron microscopy (VEGA 3, TESCAN, The Czech) according to Zhang *et al*. [42].

### Spikelet fertility and pollen analysis

Thirty days after sowing, WT and transgenic plants were transplanted in the experimental field of the Chinese National Center of Plant Gene Research (Wuhan) HUST Part (Wuhan, Hubei Province, China) with four replicates of each line (30 seedlings each replicate). Rice plants and the mature spikelets were harvested to determine spikelet fertility and weight. Pollen grains were stained with 1% I_2_-KI solution based on methods described by Zou *et al*. [69].

### Statistical analysis

Statistical analysis was carried out by both Microsoft Excel and the Statistical Package for the Social Sciences (Chicago, IL, USA). Variance analysis was conducted by comparing the statistical difference based on a Student’s *t*-test.

## Abbreviations

ABA: Abscisic acid; ABF: ABA-responsive transcription factor; CDPKs: Calcium-dependent protein kinases; SPK: Calcium-dependent seed-specific protein kinase; CBL: Calcineurin B-like; CaM: Calmodulin; DW: Dry weight; FW: Fresh weight; HSP: Heat shock protein; MDA: Malondialdehyde; *OsCPK9-OX*: *OsCPK9* overexpression; *OsCPK9*-RNAi: *OsCPK9* RNA interference; qRT-PCR: Quantitative reverse transcription-polymerase chain reaction; RWC: Relative water content; SLAC: Slow anion channel; TW: Turgid weight; WT: Wild type; VC: Vector control; WLR: Water loss rate.

## Competing interests

The authors declare that they have no competing interests.

## Authors’ contributions

GYH and GXY conceived the study. SYW, XMD, YYZ, XDL, XDZ, QCL and ZYJ performed the experiments. YL, SYZ, TS and LZW carried out the analysis. SYW, WH, GYH and GXY designed the experiments and wrote the manuscript. All authors read and approved this submitted manuscript.

## Supplementary Material

Additional file 1: Figure S1 The expression of *OsCPK9* in transgenic lines. **Figure S2.** Analysis of osmotic stress tolerance of *OsCPK9*-OX and *OsCPK9*-RNAi transgenic plants. **Figure S3.** Analysis of dehydration stress tolerance of *OsCPK9*-OX and *OsCPK9*-RNAi transgenic plants. **Figure S4.** Expression analysis of some selected ABA- and stress-responsive genes by RT-PCR analysis under no stress, ABA, or PEG6000 treatments in *OsCPK9*-OX, *OsCPK9-*RNAi, and control lines. **Table S1.** PCR primers used in qRT-PCR analysis. **Table S2.** Primer sequences used in plasmids construction and PCR. **Table S3.** Growth indices of WT, VC and positive transgenics (mean ± SE) under normal growth conditions or after osmotic treatment followed by 7 days recovery. **Table S4.** Growth indices of WT, VC and positive transgenics (mean ± SE) under normal growth or after dehydration treatment followed by 10 days recovery. **Table S5.** The number of open, closed, and partially open stomata in control plants and transgenic lines (mean ± SE) under normal conditions or drought treatment. **Table S6.** Growth indices of WT, VC and positive transgenics (mean ± SE) under normal growth and 1 μM ABA conditions. **Table S7.** Growth indices of WT, VC and positive transgenics (mean ± SE) under normal growth and 3 μM ABA conditions. Click here for file
