# Supplementary material for: A rice calcium-dependent protein kinase OsCPK9 positively regulates drought stress tolerance and spikelet fertility
Source: BMC Plant Biol. 2014 May 17;14:133. doi: 10.1186/1471-2229-14-133 (PMC4036088; doi:10.1186/1471-2229-14-133)
Supplement: Additional file 1: Figure S1 — The expression of OsCPK9 in transgenic lines. Figure S2. Analysis of osmotic stress tolerance of OsCPK9-OX and OsCPK9-RNAi transgenic plants. Figure S3. Analysis of dehydration stress tolerance of OsCPK9-OX and OsCPK9-RNAi transgenic plants. Figure S4. Expression analysis of some selected ABA- and stress-responsive genes by RT-PCR analysis under no stress, ABA, or PEG6000 treatments in OsCPK9-OX, OsCPK9-RNAi, and control lines. Table S1. PCR primers used in qRT-PCR analysis. Table S2. Primer sequences used in plasmids construction and PCR. Table S3. Growth indices of WT, VC and positive transgenics (mean ± SE) under normal growth conditions or after osmotic treatment followed by 7 days recovery. Table S4. Growth indices of WT, VC and positive transgenics (mean ± SE) under normal growth or after dehydration treatment followed by 10 days recovery. Table S5. The number of open, closed, and partially open stomata in control plants and transgenic lines (mean ± SE) under normal conditions or drought treatment. Table S6. Growth indices of WT, VC and positive transgenics (mean ± SE) under normal growth and 1 μM ABA conditions. Table S7. Growth indices of WT, VC and positive transgenics (mean ± SE) under normal growth and 3 μM ABA conditions. [file 1471-2229-14-133-S1.pdf]

Additional files

A rice calcium-dependent protein kinase OsCPK9 positively regulates drought stress tolerance and spikelet fertility

Short running title: *OsCPK9* gene function in rice

Shuya Wei[a], Wei Hu[a§], Xiaomin Deng[a§], Yingying Zhang, Xiaodong Liu, Xudong Zhao, Qingchen Luo, Zhengyi Jin, Yin Li, Shiyi Zhou, Tao Sun, Lianzhe Wang, Guangxiao Yang*, Guangyuan He*

Table S1. PCR primers used in qRT-PCR analysis

Gene	Forward primer	Reverse primer
OsbZIP23	5'-TTTGCCTTCGGAGGACTCTG-3'	5'-GGCAGATCGATCTGGCGATT-3'
Rab21	5'-CACACCACAGCAAGAGCTAAGTG-3'	5'-TGGTGCTCCATCCTGCTTAAG-3'
OsLEA3	5'- AATGATTTCCCTTTGGGTC-3'	5'- CATCAGTACACATCACCCA-3'
OsP5CS	5'-CAGAGTTGATAGTGCTGCTG-3'	5'-CAGGCTAAAAGGAGCATTTG-3'
OsNAC6	5'-CCATGGCAGCTTCCCCGGAT-3'	5'-CCAATCATCCAACCTGAGGC-3'
OsRSUS	5'-TCAACCTACTGGGACAATATTTCAC-3'	5'-GCGTTTATTTGAGGCAAGCACT-3'
OsbZIP66	5'- AACGTGGAGAAGGTGGTGG -3'	5'–TTCCTCCTGTTTCTTCTG-3'
OsbZIP72	5'- AATGAGGTAGAAGAAATGAT-3'	5'- GCACAGTCGCTGATGAAGG -3'
OsNAC9	5'-CTGAGCTACGACGATATCCA -3'	5'-GAAGAGCGACGAGTAGAAGT -3'
OsNAC45	5'-ATATGCCTCCACCTCCTCCT -3'	5'-CATCTCGTAGGGCTTTCACC -3'
OsCPK9	5'-TTGACGGCAGCGGCTACAT-3'	5'-GCTGTTGCTGAGCGTCTTG-3'
ACTIN	5'-ATGGTGGTGACGGGTGAC-3'	5'-CAGACACTAAAGCGCCCGGTA-3'

Table S2. Primer sequences used in plasmids construction and PCR

Primer	Sequence	Purpose
P1	5'-GCTCTAGAAGATCGCCATGGGCAACAC-3'	OsCPK9 amplification for rice transformation
P2	5'-CGGGATCCAATGCTGAAACAATCCCAGCGTTCA-3'	
P3	5'-GGGGTACCTTGTGCCAGATATTTGCTCC-3'	RNAi intron fragment amplification
P4	5'-GGACTAGTTGATATCCGTTCTGTTTCTA-3'	
P5	5'-GCTCTAGAACCACCGATGAGGTGGGCGC-3'	RNAi sense fragment amplification
P6	5'-GGGGTACCCGAGCTGGTACTTGTCCGTG-3'	
P7	5'-CGGGATCCACCACCGATGAGGTGGGCGC-3'	RNAi antisense fragment amplification
P8	5'-GGACTAGTCGAGCTGGTACTTGTCCGTG-3'	
P9	5'-ACGGTGTCGTCCATCACAGTTTGCC-3'	Positive plants identified
P10	5'-GGAAGTGCTTGACATTGGGGAGT-3'	

Table S3. Growth indices of WT, VC and positive transgenics (mean ±SE) under normal growth conditions or after osmotic treatment followed by 7 days recovery (*p <0.05; **p <0.01)

line	Root length (cm)		Shoot length (cm)		Fresh weight (g)		Wilted leaves (%)	Green leaves (%)
	Control	20%PEG	Control	20%PEG	Control	20%PEG	20%PEG	20%PEG
WT	5.28±0.14	5.80±0.17	21.13±0.45	18.04±1.15	0.125±0.006	0.194±0.025	77.23±5.03	22.67±4.98
VC	5.10±0.12	5.60±0.12	21.02±0.92	16.78±2.60	0.115±0.008	0.199±0.023	77.68±6.13	22.20±6.07
OE28	5.41±0.13	6.90±0.33**	20.26±0.72	22.05±1.41*	0.125±0.004	0.322±0.013**	66.68±2.98*	33.17±2.98*
OE16	5.48±0.34	7.38±0.44**	21.00±0.38	23.67±2.20*	0.110±0.005	0.271±0.049*	65.83±4.36*	30.33±6.91*
Ri16	5.05±0.16	5.57±0.18	19.84±0.50	15.87±1.24	0.108±0.004	0.174±0.015	79.50±6.87	16.67±5.43
Ri2	4.92±0.23	5.00±0.27**	19.98±0.75	13.63±1.43**	0.100±0.001	0.151±0.006*	88.33±5.27*	11.67± 5.27 *

Table S4. Growth indices of WT, VC and positive transgenics (mean ±SE) under normal growth conditions or after dehydration treatment followed by 10 days recovery (*p <0.05; **p <0.01.)

line	Root length (cm)		Shoot length (cm)		Fresh weight (mg)	
	Control	dehydration	Control	dehydration	Control	dehydration
WT	4.04±0.37	4.77±0.09	20.84±0.35	9.67±4.37	170.88±3.33	140.06±39.26
VC	4.34±0.21	5.00±0.19	19.00±0.55	8.15±3.88	165.36±2.52	146.92±20.28
OE28	4.38±0.28	6.53±0.41**	19.70±0.77	19.13±1.26**	178.20±2.27	263.10±28.36**
OE16	4.43±0.37	6.20±0.15**	20.75±1.60	21.78±1.74**	177.90±4.10	249.13±27.64*
Ri16	4.48±0.80	4.80±0.29	21.70±0.41	9.00±4.17	177.96±4.01	146.64±16.54
Ri2	4.45±0.10	4.42±0.12*	20.80±0.20	6.18±4.02	181.46±2.44	130.58±13.68

Table S5. The number of open, closed, and partially open stomata in control plants and transgenic lines under normal conditions or drought treatment.

**Stomatal status**	**Normal(number)**						**Drought(number)**					
	WT	VC	OE28	OE16	Ri16	Ri2	WT	VC	OE28	OE16	Ri16	Ri2
**Completely closed**	11.33±0.88	12±0.58	13±0.58	12.67±0.33	11±0.58	10±0.58	21±0.58	22±0.58	31±1.15**	29±1.15**	18±0.58*	16±0.58*
**Partially open**	21±1.53	21±0.58	23±0.58	22±0.58	19±0.58	18.67±0.88	24±1.00	24±1.00	24±0.58	22.67±1.20	21±0.58	19±0.58*
**Completely open**	27.67±1.45	27±0.58	24±0.58	25.33±0.67	30±0.58	31.33±1.20	15±0.58	14±0.58	5±0.58**	8.33±0.88**	21±0.58**	25±0.58**
**Total**	60	60	60	60	60	60	60	60	60	60	60	60

*p <0.05; **p <0.01.

Table S6. Growth indices of WT, VC and positive transgenics (mean ±SE) under normal growth and 1 μM ABA conditions

**line**	**Root length (cm)**		**Shoot length (cm)**		**Root dry weight (mg)**		**Shoot dry weight (mg)**	
	**Control**	**1 μM ABA**	**Control**	**1 μM ABA**	**Control**	**1 μM ABA**	**Control**	**1 μM ABA**
WT	6.88±0.17	5.98±0.14	23.46±0.53	18.89±1.46	5.75±0.05	7.80±0.34	12.93±0.69	14.28±1.54
VC	6.92±0.38	5.70±0.23	21.10±0.87	18.68±1.21	5.76±0.71	7.28±0.21	13.22±0.76	13.22±0.80
OE28	6.72±0.20	5.13±0.33**	20.46±2.26	16.11±1.66	5.78±0.29	5.65±0.21**	13.53±0.56	11.94±0.27*
OE16	6.83±0.20	5.38±0.40*	19.86±1.49	14.73±1.05**	5.85±0.28	5.48±0.27**	13.05±0.42	10.20±0.55**
Ri16	6.53±0.25	6.02±0.24	20.13±2.17	20.06±2.12	5.72±0.46	7.32±0.13	13.54±1.47	13.53±1.37
Ri2	6.48±0.39	6.34±0.17*	21.18±2.82	20.17±2.74	5.68±0.07	8.22±0.42	16.30±1.81	16.30±1.81

*p <0.05; **p <0.01.

Table S7. Growth indices of WT, VC and positive transgenics (mean ±SE) under normal growth and 3 µM ABA conditions

line	Root length (cm)		Shoot length (cm)		Root dry weight (mg)		Shoot dry weight (mg)	
	Control	3 µM ABA	Control	3 µM ABA	Control	3 µM ABA	Control	3 µM ABA
WT	5.88±0.14	3.31±0.34	19.76±0.16	7.50±0.66	7.95±0.61	5.53±0.25	19.10±1.39	6.05±0.70
VC	6.00±0.16	3.47±0.29	18.78±0.33	7.00±0.34	7.66±0.72	5.78±0.22	20.03±1.87	5.82±0.62
OE28	5.94±0.55	1.04±0.18**	19.55±0.58	3.18±0.19**	7.56±0.33	1.57±0.25**	19.72±1.05	2.67±0.41**
OE16	6.04±0.22	0.98±0.04**	20.50±0.72	4.26±0.34**	7.73±0.17	1.24±0.16**	18.16±0.59	2.67±0.35**
Ri16	6.03±0.10	3.20±0.11	20.31±0.73	6.80±0.37	7.86±0.16	6.04±0.63	19.75±0.62	6.02±0.23
Ri2	5.83±0.13	3.93±0.51	18.23±0.29	6.60±0.17	7.64±0.21	6.80±0.38**	20.37±0.69	6.13±0.40

*p <0.05; **p <0.01.

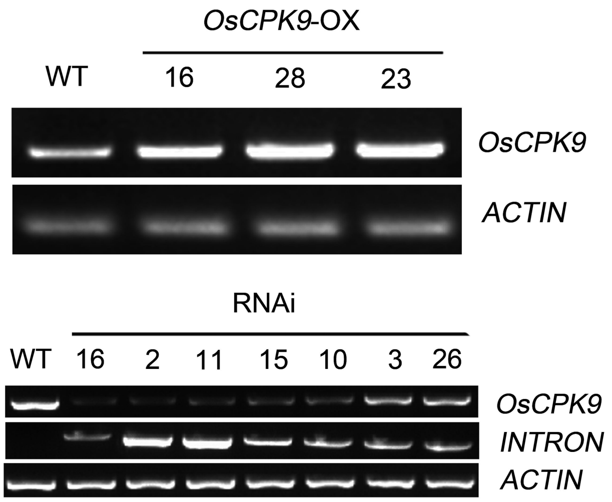

Fig. S1. The expression of *OsCPK9* in transgenic lines.

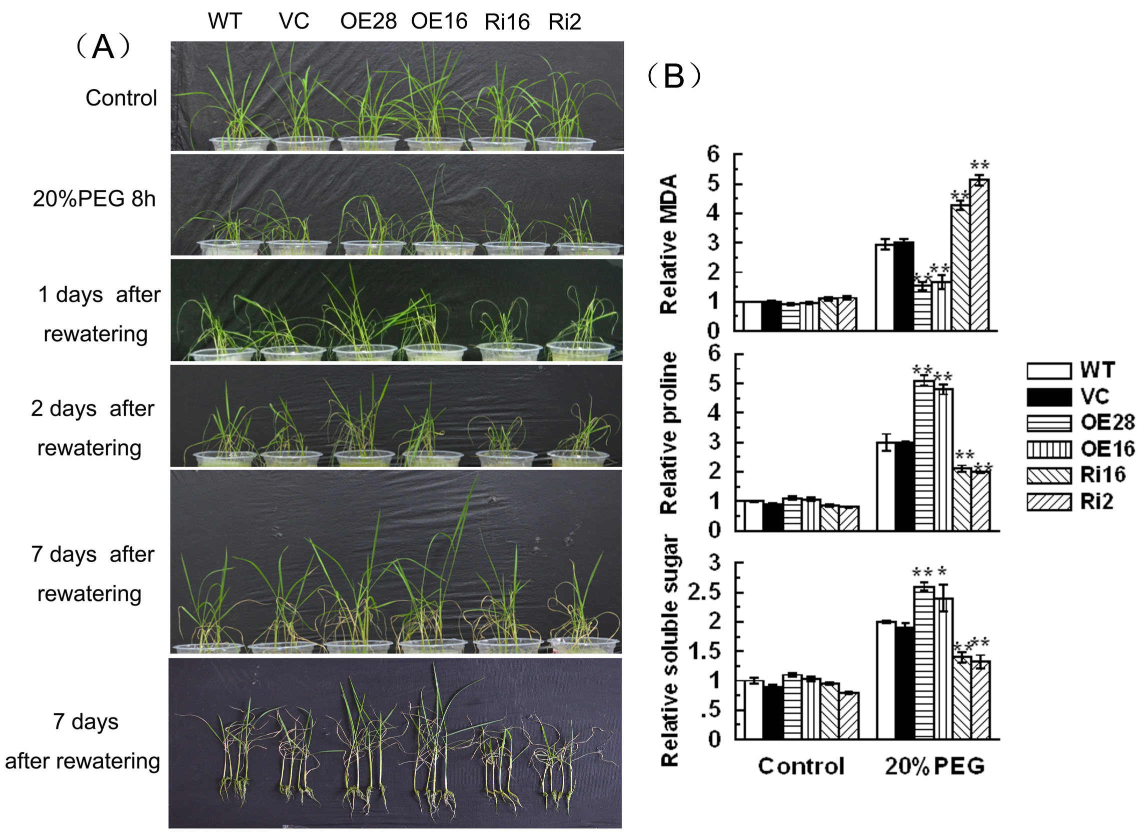

Fig. S2. Analysis of osmotic stress tolerance of *OsCPK9*-OX and *OsCPK9*-RNAi transgenic plants. Two-week-old rice seedlings were exposed to 20% PEG for 8 h and then the seedlings were recovered for 7 d to detect the performance of transgenic lines and WT (A). After PEG treatment for 8 h, leaves of rice seedling were collected

to examine MDA, proline and soluble sugar (B). Data are means ±SE calculated from four independent experiments. Asterisks indicate significant difference between the WT and transgenic lines (*p <0.05; **p <0.01).

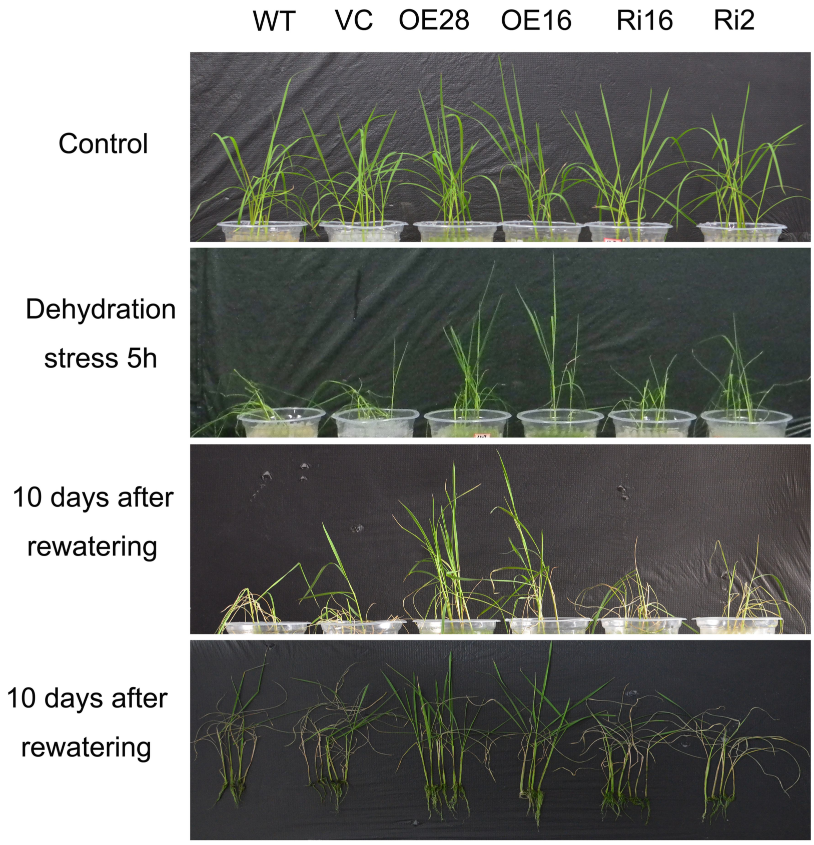

Fig. S3. Analysis of dehydration stress tolerance of *OsCPK9*-OX and *OsCPK9*-RNAi transgenic plants. Two-week-old rice seedlings were exposed to air for 5 h and then the seedlings were recovered for 10 d to detect the performance of transgenic lines and WT. Three biological experiments were conducted, which produced similar results.

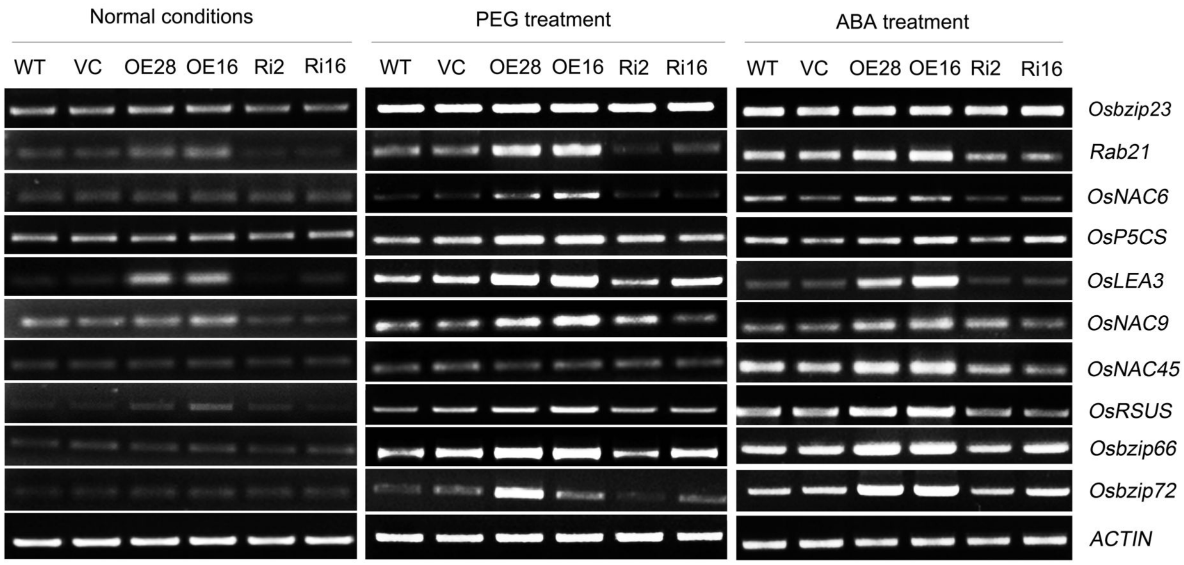

Fig. S4. Expression analysis of some selected ABA- and stress-responsive genes by RT-PCR analysis under no stress, ABA, or PEG6000 treatments in *OsCPK9*-OX, *OsCPK9* RNAi, and control lines. Three-day-old seedlings were exposed to 1 μM ABA for 14 d. Two-week-old rice plants were treated without (normal conditions) or with 20% PEG6000 for 8 h. Rice leaves were collected to detect expression of those ABA- and stress-responsive genes. Two biological experiments were performed, which produced similar results.
